# Multiscale Computational Models for Respiratory Aerosol Dynamics with Medical Applications

**DOI:** 10.1155/2019/4304139

**Published:** 2019-03-03

**Authors:** Yu Feng, Xiaole Chen, Mingshi Yang, Ke-jun Dong

**Affiliations:** ^1^School of Chemical Engineering, Oklahoma State University, Stillwater, Oklahoma, USA; ^2^School of Energy and Environment, Southeast University, Nanjing, Jiangsu, China; ^3^Department of Pharmacy, University of Copenhagen, Copenhagen, Denmark; ^4^Centre for Infrastructure Engineering, Western Sydney University, Penrith, Australia

## 1. Introduction

Inhalation of therapeutic drug aerosols is now becoming a novel way to administer micro/nanoparticles or vapors to treat lung and systemic diseases. Several attempts of such deliveries have been made at least in experimental analyses on asthma, chronic obstructive pulmonary disease (COPD), lung hypoxia, edema, lung injury, lung transplantation fungal infection, pulmonary fibrosis, and lung cancer. However, due to certain design deficiencies, existing pulmonary drug delivery devices still have poor efficiencies for delivering drugs to designated sites. Significant portions of the aggressive medicine deposit on healthy tissues, which causes severe side effects and induces extra healthcare expenses. Therefore, it is an urgent need to understand the aerosol drug dynamics better and develop a revolutionary patient-specific pulmonary drug delivery method and device to improve therapeutic outcomes by significantly improving drug delivery efficacy significantly. Due to the invasive nature and imaging resolution limitations, clinical and animal studies are not able to provide the high-resolution data for the researcher to understand the particle dynamics in human lung airways. Compared to experimental investigations, accurate and realistic computer simulation models would significantly contribute to reducing the research time and cost and visualize drug transport and translocation to multiple health endpoints via the pulmonary route.

To pave the way to developing the next-generation computational model and advance the scientific knowledge of respiratory aerosol dynamics, this special issue covers a wide range of multiscale computational models with different medical applications. Seven exciting papers are included, and they might be broadly categorized into three levels: (a) respiratory system level, (b) cell level, and (c) disease level.

## 2. Overview of the Works Published in This Special Issue

### 2.1. Respiratory System Level

The focus of the first group which consists of 3 papers, which advance the fundamental understanding on how to more realistically model the inhaled aerosol transport and deposition in human respiratory systems and how different physiological factors can influence the deposition patterns. These factors include preexisting lung disease conditions, alveolar movements, breathing patterns, and aerosol size distributions. Specifically, A. V. Kolanjiyil and C. Kleinstreuer created an elastic “whole acinar model” which covers the entire alveolated distal airways and alveolar sacs and simulates particle transport and deposition *via* CFPD coupled with the fluid-structure interaction (FSI) method. Their results indicate that the alveolar wall motion significantly increases particle deposition, and particle deposition efficiency increases with higher inhalation tidal volume and aerosol size. The acinar model can efficiently simulate aerosol dynamics in the deep lung and is ready to be incorporated into the next-generation whole-lung model. Also, focusing on the aerosol dynamics in alveolar regions, J. Xi et al. investigated the impact of Kohn structures on particle depositions in their elastic alveolar models for both healthy people and emphysema patients. Temporal and spatial deposition variations in multialveoli pore-communicated acinar models were numerically simulated. They found the size of the pores of Kohn, inhalation depth, and gravity orientation angle had insignificant effects on acinar deposition but had dramatic impact on the spatial distribution of particle deposition among alveoli. Furthermore, S. Choi et al. also used the CFPD-based model and explored the effect of altered structures and functions in severe asthma on particle deposition in subject-specific human respiratory systems. CFD results show that the induced constricted airways by asthma contribute to high wall shear stress, elevated pressure drop, and significantly increased particle deposition, compared to normal airways of healthy people.

### 2.2. Disease Level

The second group consists of 2 papers which developed mathematical models to investigate the transmission and control of infectious disease caused by inhalable bioaerosols (e.g., bacteria and virus). F. Li et al. investigated the infectious disease, e.g., the severe acute respiratory syndrome (SARS), via a stochastic susceptible-exposed-infected-quarantined-recovered (SEIQR) epidemic model with quarantine-adjusted incidence and the imperfect vaccination. Their theoretical analysis and simulations show that the stochastic disturbance is conducive to epidemic diseases control. K. Liu et al. analyzed the system dynamics of the state-dependent pulse vaccination and therapeutic strategy, which is described based on an SI ordinary differential equation model. Their results indicate that the state-dependent impulsive vaccination strategy could be used as a supplementary approach or under the situation when vaccine stockpile is limited.

### 2.3. Cell Level

The third group also consists of 2 papers, modeling transport and reproduction of cells. Y. Ji et al. studied the virus infection dynamics using a mathematical model. This model included a time delay term standing for the growth of the uninfected cells. The mathematical analysis indicates that the growth of the uninfected cells can complicate the infection results, e.g., relapse of the infection. From the biological aspect, it suggests that adequate drug treatment should be employed to avoid relapse or oscillation in the immune response. R. Wang et al. examined the transport and deformation of red blood cells through constricted microchannels using the immersed boundary-lattice Boltzmann method. Their simulations found that greater deformation and longer travel time were required to squeeze through the narrower channel.

## 3. Conclusions

This special issue documents some new studies and provides state-of-art advanced multiscale numerical modeling efforts for respiratory aerosol dynamics in pulmonary drug delivery device, human respiratory systems, and systemic regions, as well as other induced kinetics and dynamics in the human body. The accepted papers show a diversity of new findings and overviews of the recent research and development. We hope this special issue will foster a wider interest in finding the most feasible way for the development of the next-generation multiscale model, to bring the computational respiratory aerosol dynamics simulations to health endpoints with the details never undertaken before in the near future.

